# Five-year outcomes of one anastomosis gastric bypass as conversional surgery following sleeve gastrectomy for weight loss failure

**DOI:** 10.1038/s41598-022-14633-9

**Published:** 2022-06-18

**Authors:** Mohammad Kermansaravi, Reza Karami, Rohollah Valizadeh, Samaneh Rokhgireh, Ali Kabir, Mohammadali Pakaneh, Radwan Kassir, Abdolreza Pazouki

**Affiliations:** 1 Department of Surgery, Minimally Invasive Surgery Research Center, Division of Minimally Invasive and Bariatric Surgery, Rasool-E Akram Hospital, School of Medicine, Iran University of Medical Sciences, Tehran, Iran; 2 Center of Excellence of European Branch of International Federation for Surgery of Obesity, Hazrat_e Rasool Hospital, Tehran, Iran; 3 Minimally Invasive Surgery Research Center, Iran University of Medical Sciences, Tehran, Iran; 4 Department of Epidemiology, Student Research Committee, School of Public Health, Iran University of Medical Science, Tehran, Iran; 5 Endometriosis Research Center, School of Medicine, Iran University of Medical Sciences, Tehran, Iran; 6 Department of Digestive Surgery, CHU Félix Guyon, Saint Denis, La Réunion France

**Keywords:** Diseases, Gastroenterology

## Abstract

The most accepted procedures as conversion for poor weight changes after sleeve gastrectomy (SG), are malabsorptive surgeries. This study was designed to evaluate the 5-year outcomes of One Anastomosis Gastric Bypass (OAGB) following SG due to weight loss failure and weight regain. From September 2014 to January 2017, totally 23 patients with a history of SG conversion to OAGB in terms of weight loss failure or weight regain who had completed their 5-year follow-ups were studied. Some obesity related co-morbidities containing type-2 diabetes (DM), hypertension (HTN), dyslipidemia, obstructive sleep apnea (OSA) and gastroesophageal reflux disease (GERD) were also investigated at 1, 2, 3 and 5 years after conversional surgery. All cases had remission/improvement in DM, DLP, HTN and OSA 1 year after conversional OAGB. Analysis showed statistically significant (P < 0.001) change in trend of BMI. Mean BMI before conversional surgery, at 1, 2, 3and 5 years were 46.3 ± 10.4, 34.5 ± 8.5, 34.1 ± 8.6, 35.7 ± 8.7 and 37.5 ± 11.6, respectively. Mean percent excess weight loss (%EWL) at 1, 2, 3 and 5 years was 51.6 ± 11.0, 52.9 ± 13.1, 45.5 ± 16.4 and 41.0 ± 18.0, respectively. Mean percent total weight loss (%TWL) at 1, 2, 3 and 5 years was 26.6 ± 5.9, 27.4 ± 7.2, 23.9 ± 9.2 and 20.9 ± 9.3, respectively. OAGB is an effective conversional procedure for insufficient weight loss and weight regain following failed SG and lead to satisfactory changes in obesity associated medical problems. The optimal weight loss results are obtained at 2-year follow-ups and these effects are then reduced.

## Introduction

Obesity has come to a point where it is considered an epidemic problem around the world and bariatric surgery is now used as an effective solution for the treatment of obesity and its associated medical problems^1^. Nowadays, Sleeve Gastrectomy (SG) has become the most frequent bariatric surgery in the world due to the shorter duration of this procedure and its simpler technique in comparison with gastric bypass^2^. Given the rise in the number of SG operations carried out globally, studies on the long-term outcomes of this procedure have become more common.

Weight loss failure is a major reason for undergoing bariatric surgery, including SG. Some of the late complications that develop after SG include Gastroesophageal Reflux Disease (GERD), stricture, and food intolerance; these complications lead to conversion surgery in up to 25% of the patients^3^.

Various conversion procedures have been proposed for SG failure. One Anastomosis Gastric Bypass (OAGB) is a malabsorptive surgery that was first introduced by Rutledge in 1997 and is now the third most common bariatric surgery performed in the world^4^. OAGB has become a simple, feasible and effective conversion procedure for weight loss failure after SG that has satisfying effects on weight and obesity associated medical problems^5–7^ and can be safely performed in tertiary bariatric surgery centers^8^.

SG has long been performed around the world, and numerous conversion procedures have been developed for it due to various indications^1^. Some studies have shown controversial outcomes for related variables with different SG conversion procedures. There are limited published study about conversional OAGB following failed SG and most of them has reported the short to mid-term outcomes^9–11^ that should be assess in long term.

The present study was designed to evaluate the safety and 5-year outcomes of OAGB following SG due to weight loss failure and weight regain.

## Material and methods

### Patients

Data were collected from the Iranian National Obesity Surgery Database (INOSD)^12^ and all surgical procedures were performed at a tertiary, academic and accredited IFSO-EC bariatric surgery center.

**Figure 1:**
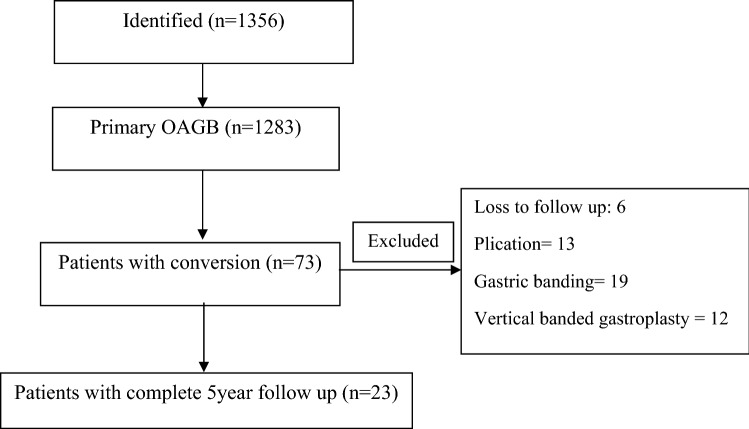
Flow chart of the patients participated in the study.

All the patients who had undergone OAGB from September 2014 to January 2017 were evaluated. During this time, 1356 patients had undergone OAGB, including 73 cases as conversion surgery. A total of 29 patients had undergone conversional OAGB following SG due to weight regain or incomplete weight loss. We only included the 23 patients who had completed their 5-year follow up (Fig. 1).

### Surgical indications

Prior to the conversional OAGB, all the patients were evaluated by a Multidisciplinary Team (MDT) and an esophagogastroduodenoscopy (EGD) was performed on them.

SG failure was taken as weight loss failure or weight regain and was defined as an unsatisfying weight loss (EWL < 50% in 1 year)^13^, a BMI greater than 35 kg/m^2^ after reaching the appropriate weight, or 25% EWL increase from the nadir weight^14^.

### Data collection

The data registered at the time of SG and OAGB included age, gender, weight, height, nadir weight, BMI, percent excess weight loss (%EWL): %EWL = [(Initial Weight) − (Post-Op Weight)]/[(Initial Weight) − (Ideal Weight)]^14^ and conversion indication.

Obesity associated medical problems included type-2 Diabetes Mellitus (DM), arterial Hypertension (HTN), dyslipidemia, Obstructive Sleep Apnea (OSA) and GERD, weight indicators and obesity associated medical problems outcomes were evaluated 1, 2, 3 and 5 years after the conversion to OAGB. The changes in obesity associated medical problems were classified in five categories: no change, remission, improvement, new onset and recurrence, which were assessed according to standardized outcomes reporting in metabolic and bariatric surgery^14^.

DM remission was defined as HbA1c < 6%, FBG < 100 mg/dl) in the absence of anti-diabetic medications. HTN remission was taken as being normotensive (BP < 120/80) without antihypertensive medications. Dyslipidemia remission was confined to normal lipid profile (LDL, HDL, Cholesterol, TG) without medication usage. GERD remission was defined as the absence of symptoms with no medications^14^ and the GERD score was assessed using the GERD-Q questionnaire^15^ in these patients. The patients with GERD score more than eight points were evaluated by EGD. DM improvement was defined as statistically significant reduction inHbA1c and FBG or decrease in antidiabetic medications requirements. HTN improvement was taken as a decrease in dosage or number of antihypertensive medications or decrease in systolic or diastolic blood pressure (BP) on the same medication. Dyslipidemia improvement was confined to decrease in number or dose of lipid-lowering agents with equivalent control of dyslipidemia or improved control of lipids on equivalent medication^14^.

### Surgical procedures

All of the surgeries were carried out by two senior surgeons of one bariatric surgery team. For these surgeries, the surgeon stands in between the patient’s legs in the French position. OAGB was performed with five trocars laparoscopic technique. The patient was administered general anesthesia. First, the His angle was released and adhesiolysis was performed. Then, the gastric pouch was constructed along the lesser curvature beginning from the distal part of the crow’s foot to the angle of His and if the sleeve tube was dilated, the pouch was trimmed on a 36 Fr calibration tube and the remnant was excised. Then, gastrojejunostomy was carried out with a 30–40 mm anastomosis length in the posterior wall of the pouch side to side with the jejunum with a biliopancreatic limb (BPL) of 180 cm for BMIs under 50 and 200 cm for BMIs of 50 and over by a linear stapler. The enterotomies were closed with one-layer absorbable suture (PDS 2-0). Finally, after obtaining a negative air leak test, a drain was placed for the patient. The average of operation time was 70 min.

### Postoperative care

On the first postoperative day, after the methylene blue leak test and clear liquid tolerance, the drain was removed and according to Enhanced Recovery after Bariatric Surgery (ERAS) protocol the patient was discharged^16^.

### Ethic issue

The research followed the tenets of the Declaration of Helsinki. The Ethics Committee of Iran University of Medical Sciences approved this study (IR.IUMS.REC.1399.801). Accordingly, written informed consent was taken from all participants before any intervention.

### Statistical analysis

The mean, standard deviation (SD), percentage and 95% confidence interval (CI) were reported for the description of the data. Repeated measurements were used to assess the trend of changes in weight, BMI, %TWL and %EWL after conversion surgery. Friedman’s test was used for changes in the pattern of obesity associated medical problems during the time. The level of statistical significance was taken as P-value < 0.05. All the analyses were carried out in SPSS version 25.0 (Chicago, Illinois, USA).

## Results

In this study, we had 73 case of conversional OAGB, 29 of which were following SG. Six cases had not complete 5-year follow up and then were excluded from the study. In the 1-year follow-up, there were no major complications and mortality to report. The major complications were defined as any complication that result in a prolonged hospital stay (beyond 7 days), re-intervention, or reoperation such as anastomotic leak requiring reoperation, venous thrombotic event (VTE) and Gastrointestinal bleeding^14^.

Mean age of the patients was 42.4 years and most of them (87.0%) were female (Table 1).

**Table 1:** Descriptive basic characteristics of patients (mean, SD, frequency and percent) (descriptive tests).

Basic characteristics	
Age (year)	42.4 ± 9.4
Female sex, n (%)	20 (87.0)
Height (cm)	163.8 ± 6.1
Weight before sleeve (kg)	145.2 ± 24.4
Nadir weight (kg)	99.7 ± 19.9
Weight before conversion (kg)	124.4 ± 30.6
BMI before sleeve (kg/m^2^)	53.8 ± 9.8
Nadir BMI (kg/m^2^)	36.8 ± 7.0
BMI before conversion (kg/m^2^)	46.3 ± 10.4
DM^a^, n (%)	4 (17.4)
Dyslipidemia^a^, n (%)	4 (17.4)
HTN^a^, n (%)	4 (17.4)
OSA^a^, n (%)	2 (8.7)
GERD^a^, n (%)	2 (8.7)
Time between sleeve and conversional surgery (year)	4.1 ± 1.8

*DM* diabetes mellitus, *GERD* gastroesophageal reflux disease, *HTN* hypertension, *No* number, *OSA* obstructive sleep apnea, *SD* standard deviation.

^a^At the time of OAGB/MGB.

The indication of conversion to OAGB was SG failure in all the cases, including 39% (n = 9) for weight regain and 61% (n = 14) for weight loss failure. The mean ± SD of the interval between SG and conversion surgery was 4.1 ± 1.8 years.

### Obesity associated medical problems outcomes

All the cases (100%) in this study had remission/improvement in DM, DLP, OSA, and HTN 1 year after their conversion surgery, but one case had DM recurrence at 5-year follow-up (Table 2). There were also 3 new-onset GERD symptoms with GERD-score more than 8 at 1-year follow-up after conversional OAGB with sign of bile reflux and esophagitis in EGD that two of them resolved at 2nd year of follow-up, but one patient had persistent GERD symptoms at 5-year follow-up and was on medication.

**Table 2:** Obesity associated medical problems at SG, at conversion to OAGB and 1, 2, 3 and 5-year follow up presented as frequency (descriptive test).

Obesity associated medical problems	At SG	At conversion to OAGB	1-year	2-year	3-year	5-year
**DM, n (%)**						
Remission	6	4	2	2	2	1
Improvement			2	2	2	2
**Dyslipidemia, n (%)**						
Remission	8	4	1	0	0	0
Improvement			3	4	4	4
**HTN, n (%)**						
Remission	5	4	3	3	3	3
Improvement			1	1	1	1
**OSA, n (%)**						
Remission	6	2	2	2	2	2
**GERD, n (%)**						
Remission	1	2	2	2	2	2
New-onset GERD	–	1	3	1	1	1

*DM* diabetes mellitus, *GERD* gastroesophageal reflux disease, *HTN* hypertension, *No* number, *OSA* obstructive sleep apnea.

### Weight changes

**Figure 2:**
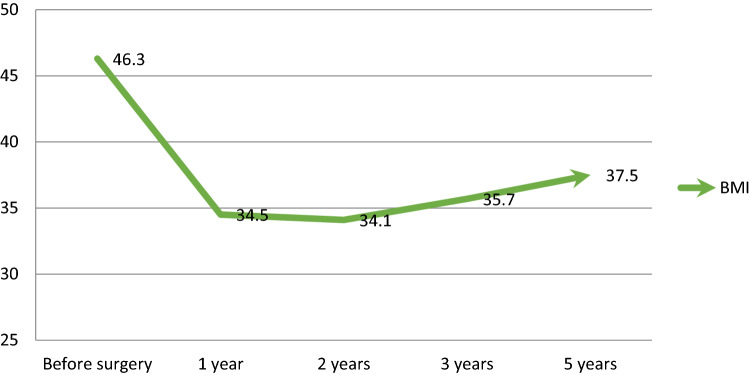
Trend of change in BMI during 5 years after conversional OAGB through repeated measure test. *BMI* body mass index, *CI* confidence interval.

**Figure 3:**
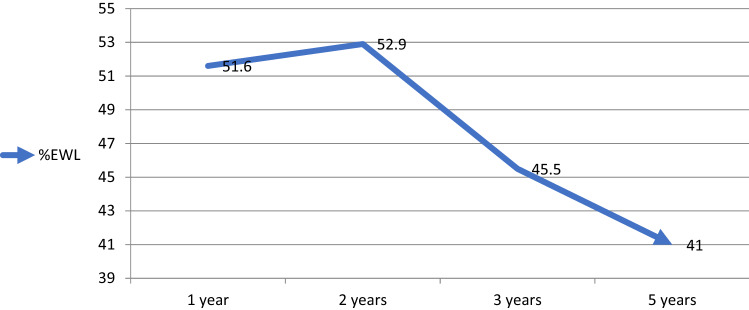
Trend of change in %EWL during 5 years after conversional OAGB through repeated measure test. *%EWL* percent excess weight loss.

The repeated measurement analysis showed statistically significant (P < 0.001) changes in the trend of BMI, which had reached its nadir 2 years after the surgery with a slow increase thereafter. Nonetheless, after 5 years, it did not reach its baseline value (Table 3, Fig. 2). The trend of changes in %EWL showed a significant (P = 0.032) decrescendo pattern after the second year following conversion surgery (Table 3, Fig. 3) as well as individual data are available in Supplementary Figs. S1 and S2. In details, Mean BMI before conversional surgery, at 1, 2, 3and 5-year follow-ups were 46.3 ± 10.4, 34.5 ± 8.5, 34.1 ± 8.6, 35.7 ± 8.7 and 37.5 ± 11.6, respectively. Mean %EWL at 1, 2, 3 and 5-year follow-ups were 51.6 ± 11.0, 52.9 ± 13.1, 45.5 ± 16.4 and 41.0 ± 18.0, respectively. Mean %TWL at 1, 2, 3 and 5-year follow-ups were 26.6 ± 5.9, 27.4 ± 7.2, 23.9 ± 9.2 and 20.9 ± 9.3, respectively (Table 3).

**Table 3:** Trend of change in BMI, %EWL and %TWL during 5 years after conversional surgery (using parametric repeated measure ANOVA; P value < 0.001 for BMI, P value-0.008 for TWL and P value = 0.032 for EWL).

Variable	Before surgery^a^	1 year^b^	2 years^b^	3 years^b^	5 years^b^
BMI (kg/m^2^)	46.3 ± 10.4	34.5 ± 8.5	34.1 ± 8.6	35.7 ± 8.7	37.5 ± 11.6
EWL (%)	–	51.6 ± 11.0	52.9 ± 13.1	45.5 ± 16.4	41.0 ± 18.0
TWL (%)	–	26.6 ± 5.9	27.4 ± 7.2	23.9 ± 9.2	20.9 ± 9.3

*BMI* body mass index, *EWL* excess weight loss, *TWL* total weight loss.

Values are mean ± standard deviation.

^a^Just before conversional surgery.

^b^After surgery.

## Discussion

The rate of conversion surgery, especially conversional surgeries following SG has increased in the last years at bariatric surgery centers According to the IFSO global registry report, SG is the most common bariatric intervention worldwide^2^. The trends show that SG is largely performed due to its relatively simple technique, shorter operation time and satisfactory short and long-term outcomes. Given the rapidly increasing number of patients who have undergone SG, the mid and long-term outcomes of this procedure including weight loss outcomes and GERD should evaluate more. A mean percent excess weight loss (EWL) of 50–60% is expected in the long term after SG^17^. The most common indication for SG conversion is weight loss failure and weight regain, which are reported in up to 18% of the cases in recent series^18^. The other relatively common post-SG complication is GERD with unknown exact mechanism and long-term incidence about 20–30% and its relationship with Barrett’s esophagus^17^. These complications may be treated by SG conversion to other procedures such as RYGB, biliopancreatic diversion with duodenal switch (BPD-DS) and OAGB^19^.

Conversion surgeries after SG are relatively prevalent worldwide. The most accepted procedures as conversion for poor weight changes after SG include malabsorptive surgeries such as BPD-DS or OAGB^8^. In comparison with RYGB and BPD-D, due to the simpler technique and shorter operation time, OAGB has been more popular worldwide. Its postoperative complications are acceptable and make this conversion a relatively safe procedure^7^. Given the longer biliopancreatic limb (BPL) than RYGB, more malabsorption and weight loss is expected.

In recent study, the interval between SG and conversional OAGB was 4.1 ± 1.8 years. Rayman et al. reported a mean interval of 5.6 years between two bariatric surgical procedures^9^.

Many studies have demonstrated the efficacy of OAGB on weight loss and obesity associated medical problems as a primary bariatric procedure. A multicenter study showed an EWL of 77% in a 5-year follow-up after OAGB^20^. Two recently-published systematic reviews showed that OAGB is a valuable choice as a conversion bariatric surgery following a failed restrictive bariatric procedure^21,22^. Our study showed mean BMI, EWL% and %TWL of 34.5 ± 8.5 kg/m^2^, %51.6 ± 11.0 and %26.6 ± 5.9 at 1-year follow-up after conversional OAGB respectively. Gerges et al. in a similar study on 28 patients after conversional OAGB resulted in 79% and 31.7% 1-year EWL% and TWL% respectively^10^. Bhandari et al. showed 1-year mean BMI, mean EWL% and mean TWL% of 34.3 kg/m^2^, 54.9% and 22% respectively^5^. Another similar study by Debs et al. showed 1-year BMI, EWL% and TWL% of 29.8 kg/m^2^, 74% and 25%^23^. Poghosyan et al. reported mean BMI, mean EWL% and mean TWL% of 34.6 kg/m^2^, 60% and 29% at 1-year follow-ups respectively^7^.

The present study found that the highest EWL% pertained to the second year after conversion and was 52.9 ± 13.1%, and the trend of changes was significant (P = 0.032). Similar to present study, Debs et al. reported the same results and showed that maximum weight loss can be reached at 2-year follow-up^23^. Poghosyan et al. showed that the best weight loss outcomes are achieved at 3-year follow-up after conversion of SG to OAGB and will decrease after that^7^. Bhandari et al. showed that weight loss continues until 2 years after conversional OAGB and there is a tendency towards weight regain and recurrence of obesity associated medical problems after 3 years^5^. They reported recurrence of DM in one patients at 3-year follow-up, similar to recent study that we found DM recurrence in one patient at 5-year follow-up.

In terms of surgical technique, the conversion of SG to OAGB is more feasible and simple than RYGB or BPD/DS.

The desirable effects of conversional OAGB on obesity associated medical problems have been previously reported. The present study demonstrated that all the patients with DM, DLP, HTN and OSA experienced improvements or remission in the first year following conversional OAGB. These results are comparable with the findings reported by Poghosyan et al., who examined 72 patients after OAGB as conversion following SG and showed 80% improvement in DM and 70% in OSA^7^. In another study by Bhandari et al., the rate of remission was 100% for DM and HTN at 1 and 2-year follow-ups^5^. Another study showed remission of DM, HTN, DLP and GERD in 91.6%, 64.7%, 70.5% and 80% of patients at 1-year follow-up respectively^11^. Debs et al., showed the improvement/remission of DM, OSA and HTN in 76.9%, 81.8% and 82.6% of patients in 1–5 years follow-ups after conversion of SG to OAGB^23^. These findings show that OAGB has significant effects on obesity associated medical problems as a conversional surgery after SG and leads to satisfactory outcomes.

Despite the efficacy and safety of conversional OAGB, there is a concern around new-onset GERD. Our study showed 3 new-onset GERD (13%) at 1-year follow-up. Debs et al. reported new-onset GERD in 9% of patients after conversional OAGB^23^. Poghosyan et al. reported the 8.3% de novo GERD after conversion of SG to OAGB, that most of them needs conversion to RYGB^7^. Rayman et al. compared the efficacy of RYGB to OAGB as conversional surgery after SG failure. They reported GERD in 17.4% patients after conversion of SG to OAGB and resulted that despite better weight loss outcomes of conversional OAGB, the incidence of GERD and nutritional deficiencies are more common in conversional OAGB compare to conversional RYGB^9^. Therefore, it can be concluded that OAGB has different effects on GERD, although some patients get symptom-free and most symptomatic patients will experience improvements by medication administration^15^.

This study presents some limitations. It was a retrospective study however; data were collected through a prospective search in on-line database. Relatively low sample size despite the 5-year follow-up period was another limitation of study.

## Conclusion

OAGB is an effective and safe conversional bariatric surgical procedure for insufficient weight loss and weight regain following failed SG that also can lead to satisfactory changes in obesity associated medical problems. The optimal weight loss results will be obtained at 2-year follow-ups and these effects are then reduced.

## Supplementary Information

Supplementary Information.

## Data Availability

The data that support the findings of this study are available from Iran National Obesity Surgery Database (INOSD) but restrictions apply to the availability of these data, which were used under license for the current study, and so are not publicly available. Data are however available from the authors upon reasonable request and with permission of INOSD.

## References

[1] Altieri MS (2018). Rate of revisions or conversion after bariatric surgery over 10 years in the state of New York. Surg. Obes. Relat. Dis..

[2] Welbourn R (2019). Bariatric surgery worldwide: Baseline demographic description and one-year outcomes from the fourth IFSO global registry report 2018. Obes. Surg..

[3] Arman GA (2016). Long-term (11+ years) outcomes in weight, patient satisfaction, comorbidities, and gastroesophageal reflux treatment after laparoscopic sleeve gastrectomy. Surg. Obesity Relat. Diseases.

[4] Angrisani L (2018). IFSO worldwide survey 2016: Primary, endoluminal, and revisional procedures. Obes. Surg..

[5] Bhandari M (2019). Revision operation to one-anastomosis gastric bypass for failed sleeve gastrectomy. Surg. Obes. Relat. Dis..

[6] Kapoulas S, Sahloul M, Singhal R (2021). Laparoscopic conversion of sleeve gastrectomy to one anastomosis gastric bypass in a hostile abdomen. Obes. Surg..

[7] Poghosyan T (2019). Conversion of sleeve gastrectomy to one anastomosis gastric bypass for weight loss failure. Obes. Surg..

[8] Musella M (2019). Conversion from laparoscopic adjustable gastric banding (LAGB) and laparoscopic sleeve gastrectomy (LSG) to one anastomosis gastric bypass (OAGB): Preliminary data from a multicenter retrospective study. Surg. Obes. Relat. Dis..

[9] Rayman S (2021). Sleeve gastrectomy failure-revision to laparoscopic one-anastomosis gastric bypass or Roux-n-Y Gastric Bypass: A multicenter study. Obes. Surg..

[10] Gerges WB, Omran H, Makram F (2022). Conversion of laparoscopic sleeve gastrectomy after weight loss failure into laparoscopic one anastomosis gastric bypass: Short-term safety and efficacy and effect of indications on outcome. Surg. Endosc..

[11] Sabry AA, Mohamed HM, Helmy MM, Elshimy MA (2020). Laparoscopic mini gastric bypass as a revisional procedure after failed primary restrictive bariatric surgery. Ain Shams Med. J..

[12] Kermansaravi M (2022). The First web-based Iranian national obesity and metabolic surgery database (INOSD). Obes. Surg..

[13] Sepúlveda M (2017). Long-term weight loss in laparoscopic sleeve gastrectomy. Surg. Obesity Relat. Diseases.

[14] Brethauer SA (2015). Standardized outcomes reporting in metabolic and bariatric surgery. Surg. Obes. Relat. Dis..

[15] Kermansaravi M, Kabir A, Mousavimaleki A, Pazouki A (2020). Association between hiatal hernia and gastroesophageal reflux symptoms after one-anastomosis/mini gastric bypass. Surg. Obes. Relat. Dis..

[16] Małczak P (2017). Enhanced recovery after bariatric surgery: Systematic review and meta-analysis. Obes. Surg..

[17] Chiappetta S, Stier C, Scheffel O, Squillante S, Weiner RA (2019). Mini/one anastomosis gastric bypass versus Roux-en-Y gastric bypass as a second step procedure after sleeve gastrectomy—A retrospective cohort study. Obes. Surg..

[18] Moon RC, Fuentes AS, Teixeira AF, Jawad MA (2019). Conversions after sleeve gastrectomy for weight regain: To single and double anastomosis duodenal switch and gastric bypass at a single institution. Obes. Surg..

[19] Carmeli I, Golomb I, Sadot E, Kashtan H, Keidar A (2015). Laparoscopic conversion of sleeve gastrectomy to a biliopancreatic diversion with duodenal switch or a Roux-en-Y gastric bypass due to weight loss failure: our algorithm. Surg. Obes. Relat. Dis..

[20] Musella M (2014). The laparoscopic mini-gastric bypass: The Italian experience: Outcomes from 974 consecutive cases in a multicenter review. Surg. Endosc..

[21] Kermansaravi M (2021). One anastomosis/mini-gastric bypass (OAGB/MGB) as revisional surgery following primary restrictive bariatric procedures: A systematic review and meta-analysis. Obes. Surg..

[22] Parmar CD (2020). One Anastomosis/Mini Gastric Bypass (OAGB-MGB) as revisional bariatric surgery after failed primary adjustable gastric band (LAGB) and sleeve gastrectomy (SG): A systematic review of 1075 patients. Int. J. Surg..

[23] Debs T (2020). Laparoscopic conversion of sleeve gastrectomy to one anastomosis gastric bypass for weight loss failure: Mid-term results. Obes. Surg..

