# Supplementary material for: Five-year outcomes of one anastomosis gastric bypass as conversional surgery following sleeve gastrectomy for weight loss failure
Source: Sci Rep. 2022 Jun 18;12:10304. doi: 10.1038/s41598-022-14633-9 (PMC9206653; doi:10.1038/s41598-022-14633-9)
Supplement: Supplementary file 1 — Supplementary Information. [file 41598_2022_14633_MOESM1_ESM.docx]

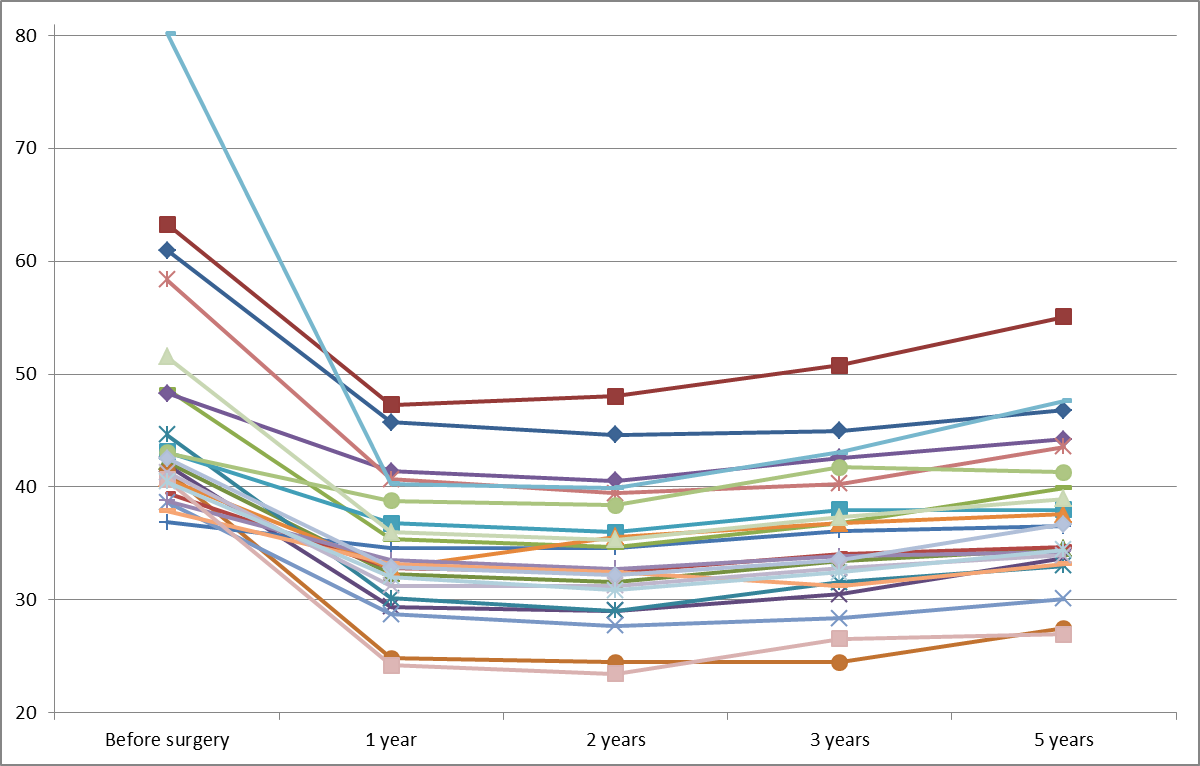


Figure S1 - Trend of change in body mass index (Kg/m^2^) during 5 years after conversional OAGB for each individual

Figure S2 - Trend of change in excess weight loss during 5 years after conversional OAGB for each individual
